# Prognostic Value of Procalcitonin and C-Reactive Protein in 1608 Critically Ill Patients with Severe Influenza Pneumonia

**DOI:** 10.3390/antibiotics10040350

**Published:** 2021-03-26

**Authors:** Raquel Carbonell, Gerard Moreno, Ignacio Martín-Loeches, Frederic Gomez-Bertomeu, Carolina Sarvisé, Josep Gómez, María Bodí, Emili Díaz, Elisabeth Papiol, Sandra Trefler, Mercedes Nieto, Angel Estella, María Jiménez Herrera, Pablo Vidal Cortés, Juan José Guardiola, Jordi Solé-Violán, Alejandro Rodríguez

**Affiliations:** 1 Critical Care Department, Hospital Universitari Joan XXIII, 43005 Tarragona, Spain; murenu77@hotmail.com (G.M.); sitrefler@yahoo.es (S.T.); 2 Department of Anaesthesia and Critical Care, St James’s University Hospital, Trinity Centre for Health Sciences, Multidisciplinary Intensive Care Research Organization (MICRO), Dublin 8, Ireland; drmartinloeches@gmail.com; 3 Microbiology, Hospital Universitari Joan XXIII, 43005 Tarragona, Spain; ffgomez.hj23.ics@gencat.cat (F.G.-B.); csarvise.tgn.ics@gencat.cat (C.S.); 4 Tarragona Health Data Research Working Group (THeDaR)-ICU Hospital Joan XXIII, 43005 Tarragona, Spain; josep.goal@gmail.com; 5 Critical Care Department URV/IISPV/CIBERES, Hospital Universitari Joan XXIII, 43005 Tarragona, Spain; mbodi.hj23.ics@gencat.cat (M.B.); ahr1161@yahoo.es (A.R.); 6 Critical Care Department/CIBERES, Hospital Parc Taulí, 08208 Sabadell, Spain; emilio.diaz.santos@gmail.com; 7 Critical Care Department, Hospital Universitari Vall d’Hebron, 08035 Barcelona, Spain; elisabeth_papiol@hotmail.com; 8 Critical Care Department, Hospital Clínico San Carlos, 28040 Madrid, Spain; mernietoc@gmail.com; 9 Critical Care Department, Hospital de Jerez, 11407 Jerez de la Frontera, Spain; litoestella@hotmail.com; 10 Dean Nursing Faculty, Universitat Rovira i Virgili, 43003 Tarragona, Spain; maria.jimenez@urv.cat; 11 Critical Care Department, Complejo Hospitalario Universitario Ourense, 32005 Ourense, Spain; pablomopvc@hotmail.com; 12 Department of Pulmonary, Critical Care and Sleep Medicine, University of Louisville, Louisville, KY 40202, USA; juan.guardiola@va.gov; 13 Critical Care Department Hospital Universitario Dr. Negrín, 35010 Las Palmas de Gran Canaria, Spain; jsolvio@gobiernodecanarias.org

**Keywords:** pneumonia, procalcitonin, C-Reactive protein, mortality, Gram-positive cocci, Gram negative bacilli

## Abstract

*Background*: Procalcitonin (PCT) and C-Reactive protein (CRP) are well-established sepsis biomarkers. The association of baseline PCT levels and mortality in pneumonia remains unclear, and we still do not know whether biomarkers levels could be related to the causative microorganism (GPC, GNB). The objective of this study is to address these issues. *Methods*: a retrospective observational cohort study was conducted in 184 Spanish ICUs (2009–2018). *Results*: 1608 patients with severe influenza pneumonia with PCT and CRP available levels on admission were included, 1186 with primary viral pneumonia (PVP) and 422 with bacterial Co-infection (BC). Those with BC presented higher PCT levels (4.25 [0.6–19.5] *versus* 0.6 [0.2–2.3]ng/mL) and CRP (36.7 [20.23–118] *versus* 28.05 [13.3–109]mg/dL) as compared to PVP (*p* < 0.001). Deceased patients had higher PCT (ng/mL) when compared with survivors, in PVP (0.82 [0.3–2.8]) *versus* 0.53 [0.19–2.1], *p* = 0.001) and BC (6.9 [0.93–28.5] *versus* 3.8 [0.5–17.37], *p* = 0.039). However, no significant association with mortality was observed in the multivariate analysis. The PCT levels (ng/mL) were significantly higher in polymicrobial infection (8.4) and GPC (6.9) when compared with GNB (1.2) and *Aspergillus* (1.7). The AUC-ROC of PCT for GPC was 0.67 and 0.32 for GNB. The AUROC of CRP was 0.56 for GPC and 0.39 for GNB. *Conclusions*: a single PCT/CRP value at ICU admission was not associated with mortality in severe influenza pneumonia. None of the biomarkers have enough discriminatory power to be used for predicting the causative microorganism of the co-infection.

## 1. Introduction

The diagnosis of sepsis in critically ill patients is challenging, because sepsis response is complex. Although bacterial culture is the best method for diagnosing infection, it does not predict mortality, it may require more than 24 h for confirmation, and some critically ill patients may have received prior antimicrobial therapy, which can render negative microbial cultures [1,2]. Thus, biomarkers of inflammation or infection, such as procalcitonin (PCT), C-reactive protein (CRP), interleukin-6 (IL-6), neopterin, and pro-adrenomedullin (pro-ADM), have a significant role in the diagnosis and management of sepsis [3,4]. CRP and PCT are currently the most frequently used biomarkers of sepsis in clinical practice.

CRP is a well-established marker of inflammation, but it has been considered to be insufficient as a useful biomarker to predict prognosis and microbial patterns in sepsis [5]. PCT is assumed to be a superior marker; several studies have shown that PCT is highly sensitive and specific for early distinguishing non-infectious systemic inflammatory response syndrome (SIRS) from sepsis [6]. PCT concentration decreases under antibiotic therapy; hence, it can adequately monitor infection evolution with adequate therapy and can help to adjust antibiotic duration [7,8]. Moreover, persistently high serum PCT levels correlate with the severity of systemic infection in sepsis and septic shock [9,10]. However, the role of initial serum PCT levels on intensive care unit (ICU) admission to predict outcome in patients with sepsis is unclear. 

In addition, the association of PCT and microbial etiology has been a matter of debate. Several studies have reported higher PCT levels in Gram-negative bacteremia as compared to Gram-positive bacteremia and fungal infection [11,12]. Nevertheless, most of these studies are based on sepsis with different sources of infection and, therefore, with different predominant microorganism, which could influence the results.

Our hypothesis was that PCT can be a helpful biomarker to determine prognosis and predict microbial etiology in patients with influenza admitted to ICU. Therefore, the aim of the present study was to evaluate the association of baseline serum PCT and CRP levels and mortality, among patients with sepsis and septic shock, with a respiratory source of infection. In addition, we evaluated the performance of both biomarkers to predict causative pathogens of the infection.

## 2. Results

**Figure 1:**
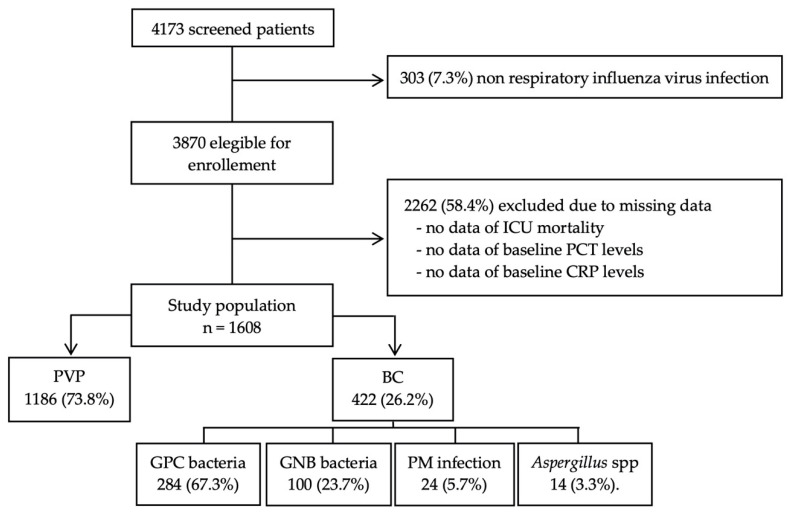
Flow diagram of patient enrollment. Abbreviations: ICU, Intensive Care Unit; PCT, procalcitonin; CRP, C-Reactive protein; PVP, primary viral pneumonia; BC, bacterial co-infection; GPC, Gram Positive bacteria cocci; GNB, Gram Negative bacteria bacilli; PM, polymicrobial.

A total of 4173 patients with influenza infection were enrolled at 184 ICUs between the study period; 2262 patients were excluded because of missing values in PCT and CRP at admission or in mortality, and 303 were excluded because of the non-respiratory source of infection. Finally, 1608 patients met the inclusion criteria and were included in the current analysis, 1186 (73.8%) patients with primary viral pneumonia (PVP), and 422 (26.2%) with bacterial co-infection (BC) (Figure 1).

Table 1 details the main demographic and clinical characteristics of patients. The median age was 56 years (IQR 46–67) and 60% were male. The patients presented a median Acute Physiology and Chronic Health Evaluation (APACHE) II and Sequential Organ Failure Assessment (SOFA) scores of 17 (IQR 12–22) and 6 (IQR 4–9), respectively. The most frequent comorbidity was chronic obstructive pulmonary disease (20.4%). 32.9% (*n* = 530) developed acute kidney failure, 57.1% (*n* = 920) presented septic shock at ICU admission, and 81.9% (*n* = 1318) required mechanical ventilation (MV) (58% invasive and 23.9% non-invasive MV).

### 2.1. Comparison Between Patients with and Without Bacterial Co-Infection

Patients with BC were sicker when compared to PVP, showing higher APACHE II (19 [IQR 14–25] *versus* 16 [IQR 11–21], *p* = 0.001) and SOFA scores (7 [IQR 4–10] *versus* 6 [IQR 4–8], *p* = 0.001), were more likely to present with shock (67.3% *versus* 53.5%, *p* = 0.001), had higher rate of acute kidney injury (45.7% *versus* 28.4%, *p* = 0.001), and needed more renal replacement therapy (17.8% *versus* 11.8%, *p* = 0.002). Patients with BC more frequently had asthma (29.4% *versus* 17.1%, *p* = 0.001), and they had a lower proportion of obesity (5.5% *versus* 34.3%, *p* = 0.01). No differences in another comorbidity, gender, duration of MV, or ICU length of stay were observed. The patients with co-infection had higher median PCT (4.35 [IQR 0.6–19.5] *versus* 0.6 [IQR 0.2–2.3] ng/mL, *p* = 0.001) and CRP levels (36.7 [IQR 20.23–118] *versus* 28.05 [IQR 13.3–109] mg/dL, *p* = 0.001]) when compared with PVP. Table 1 shows the complete characteristics.

### 2.2. Performance of PCT or CRP as Predictors of the Microorganism Responsible of Bacterial Co-Infection

Among 1608 patients included, 408 had confirmed bacterial coinfection and 14 *Aspergillus* spp. isolation and were included in the present analysis. Gram Positive cocci (GPC) bacteria were isolated in 67.3% (*n* = 284) and Gram Negative bacilli (GNB) bacteria in the 23.7% (*n* = 100) of patients. Twenty-four episodes (5.7%) were polymicrobial (PM) infections and *Aspergillus* spp. was isolated in 14 patients (3.3%). Table 1 details the main demographic and clinical characteristics of patients.

Among patients with co-infection, the overall ICU mortality was 27.7% (*n* = 117). The highest mortality rate was observed in the *Aspergillus* spp. group (50%), being higher than in GPC (25%, *p* = 0.03).

**Figure 2:**
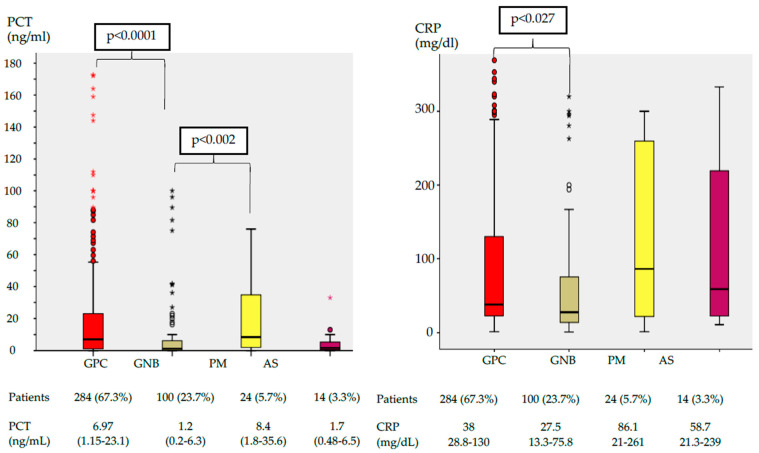
Box plot of serum PCT and CRP concentration by pathogen group. The center of each box plot represents the median, with the box denoting the IQR, the whiskers representing 1.5 times the IQR, and dots showing outliers beyond the whiskers. Data are expressed as numbers (%) and medians (IQR). Abbreviations: PCT, procalcitonin; CRP, C-Reactive protein; GPC, Gram Positive bacteria cocci; GNB, Gram Negative bacteria bacilli; PM, polymicrobial; AS, *Aspergillus*; IQR, interquartile range.

The serum PCT levels were significantly higher for PM (8.4 ng/mL) and GPC infections (6.9 ng/mL) than for patients with GNB (1.2 ng/mL) and *Aspergillus* spp. (1.7 ng/mL). The highest CRP concentrations were found in PM (86.1 mg/dL) (Figure 2).

Figure S1 (Supplementary Material) details the levels of PCT and CRP according to different microorganisms.

The corresponding ROC curve was constructed in order to assess whether biomarkers could differentiate between GPC and GNB infection. The AUC-ROC of PCT for GPC was 0.67 (95% CI, 0.61–0.73, *p* < 0.001) and 0.32 (95% CI, 0.26–0.38, *p* < 0.001) for GNB bacteria (Figure S2, Supplementary Material). The different cut-off values for PCT are shown in Table S1 (Supplementary Material). The optimal cut-off value of PCT to predict GPC infection was 10.18 ng/mL, with a sensitivity of 42%, a specificity of 83%, a positive predictive value of 83%, and a negative predictive value of 34%. The AUC-ROC of CRP was 0.56 (95% CI, 0.50–0.62, *p* = 0.04) for GPC and 0.39 (95% CI, 0.32–0.45, *p* < 0.01) for GNB respiratory infection.

### 2.3. Mortality Analysis 

The overall ICU mortality was 23.8 % (*n* = 382), and it was significantly higher in the patients with co-infection as compared to PVP (27.7% *versus* 22.3%, *p* = 0.02). The characteristics of patients according to ICU outcome are shown in the Supplementary Material (Table S2). Severity of illness (APACHE II, SOFA) were significantly higher in non-survivors. In addition, non-survivors developed more complications, such as acute kidney injury, shock at ICU admission, and required more days of MV. 

**Figure 3:**
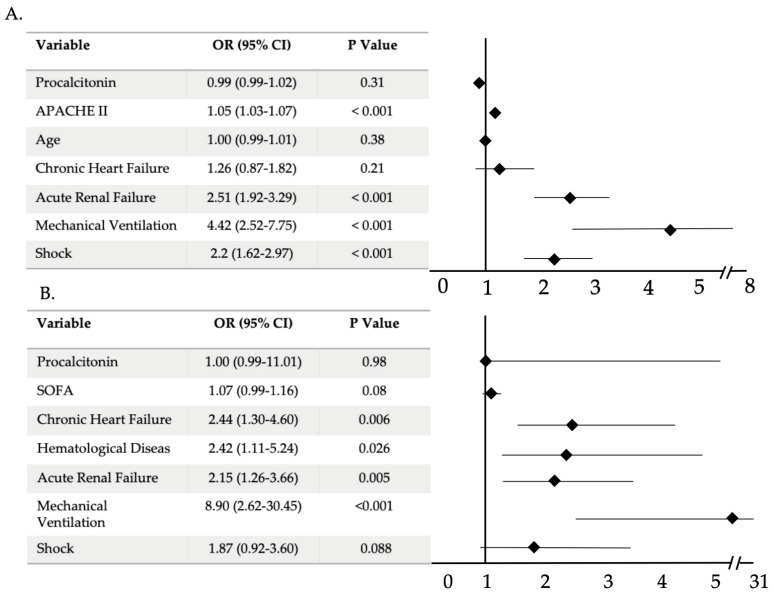
Multivariable analysis of factors associated with ICU mortality in overall population (**A**) and in bacterial co-infection group (**B**). Abbreviations: APACHE, Acute Physiology and Chronic Health Evaluation; SOFA, sequential organ failure assessment; CI, confidence interval.

Non-survivors had significantly higher levels of PCT in both groups: PVP (0.82 [IQR 0.3–2.8]) *versus* 0.53 ng/mL [IQR 0.19–2.1], *p* = 0.001) and BC (6.9 [IQR 0.93–28.5] *versus* 3.8 ng/mL [0.5–17.37], *p* = 0.039) than survivors. However, the CRP levels did not show any significant difference between both groups. Nevertheless, in the multivariable logistic regression analysis, PCT and CRP levels at ICU admission were variables not independently associated with ICU mortality in overall population or in the BC group (Figure 3).

## 3. Materials and Methods

### 3.1. Study Design

This was a sub-analysis of a multicenter and retrospective study conducted in 184 ICUs in Spain between June 2009 and April 2018. The data were obtained from a registry created by SEMICYUC (Spanish Society of intensive care). The information was collected by the attending clinician using a paper case report form (Supplementary material).

The Ethics Committee Board of the Joan XXIII Hospital approved the study (IRB#11809). All of the data were de-identified; therefore, the requirement for informed consent was waived. The clinical management for all critical ill patients was left to the attending clinician, following the recommendations of the SEMICYUC [13]. 

To evaluate the prognostic value of biomarkers, we included all of the patients admitted to adult ICU (over fifteen years) with pneumonia due to influenza virus, with or without bacterial co-infection, in whom PCT and CRP were determined upon admission. We only considered those cases of influenza pneumonia with bacterial or *Aspergillus* spp. co-infection in order to evaluate the role of biomarkers to predict the microbial etiology of infection. We excluded patients with missing data regarding serum biomarkers levels or ICU-mortality.

In a post hoc analysis to evaluate the statistical power of the study, a sample size of 420 patients for each group (primary viral pneumonia and bacterial coinfection) was required to identify an absolute difference of 2.5 ng/mL in PCT levels in patients with coinfection with a power of 0.8 (two-tailed) at a level of significance of 0.05. The post hoc study power that was calculated with 1608 patients included was 100% with a safety level 0.95 and typical deviation of 3.6.

### 3.2. Data Collection and Laboratory Diagnostics

Data that were collected at ICU admission included demographic and general characteristics, underlying diseases, microbiological results, laboratory data, and complications during ICU stay. The severity of illness was evaluated by the APACHE II score [14] calculated for all patients within the first 24 hours of ICU admission and organ failure was assessed using the SOFA score [15].

PCT and CRP measurements were performed in local laboratories as a part of routine clinical care. PCT was measured using B·R·A·H·M·S PCT automated immunoassays. The analytical sensitivity of all assays was < 0·05 µg/L. All of the commercial quantitative BRAHMS PCT assays use the same ‘sandwich ELISA’ principle to quantify procalcitonin by forming antibody–procalcitonin–antibody complexes. The mechanism of detection of these complexes is the main difference between these assays. CRP was determined using a standardized scattering turbidimetric assay from different manufactures according to each hospital. CRP in a reference population is heavily skewed towards the detection limits of even highly sensitive assays. The quoted upper reference limits vary depending on assay, but they are typically between 3 and 10 mg/L.

Criteria for etiological diagnosis of co-infection from respiratory cultures were defined when pathogens were isolated from quantitative samples of lower respiratory tract. Significant cut-offs were considered when the growth of bacteria was ≥10^5^ colony forming units (cfu)/mL and ≥10^4^ cfu/mL for trachea-bronchial aspirates and for bronchoalveolar lavage, respectively [16]. In the case of isolation of two pathogens in respiratory sample, we only considered polymicrobial infection if both of the pathogens presented the mentioned significant threshold. In non-ventilated patients, conventional sputum was the most common respiratory sample obtained. Because of its lower sensitivity value, good quality of the sputum (defined as purulent sputum showing less than ten epithelial cells per 100X field and more than 25 leukocytes per 100× field) was required to consider a microorganism as the causative pathogen of co-infection.

Regarding to co-infection, the isolated pathogens were divided into GPC, GNB, PM infection, and *Aspergillus* spp. The serum levels of both biomarkers were also determined for the most frequent microorganisms.

### 3.3. Study Definitions

Primary viral pneumonia (PVP) was defined as acute respiratory failure with suggestive signs and symptoms of a lower respiratory tract infection, pulmonary infiltrates involving at least two lung lobes on the chest X-ray and positive rt-PCR test for influenza virus, and negative bacterial cultures at ICU admission [17].

Bacterial Co-infection (BC) was diagnosed with one or more positive bacterial or fungal blood or respiratory cultures, and/or positive urinary antigens (*Streptococcus pneumoniae* and *Legionella pneumophila*) within the first two days of hospital admission [18,19].

Polymicrobial (PM) infections: BC that is caused by two or more infectious agents [20].

The Supplementary Material details other definitions.

### 3.4. Endpoints 

The primary endpoint of our study was to evaluate whether there is an association between serum PCT and CRP levels at ICU-admission and crude ICU mortality. The secondary outcome was to analyze whether either PCT or CRP can predict the causative pathogens that are responsible for the bacterial coinfection.

### 3.5. Statistical Analysis

Categorical variables were reported as numbers (%) and differences between groups were assessed using the Chi-square test and Fisher’s exact test. Continuous variables were expressed as medians and interquartile range (IQR), and differences between groups were compared while using Student’s *t* test or Mann–Whitney U test. A logistic regression analysis was conducted to identify variables associated with ICU-mortality. The variables included in the model were those with statistical significance in the univariate analysis and those with clinical relation with the dependent variable.

Sensitivity, specificity, positive predictive value, negative predictive value, and receiver operating characteristic (ROC) with area under the curve (AUC) were calculated to investigate the diagnostic test performance for PCT and CRP to predict Gram-negative or Gram-positive infection. In a second step, optimal cut-off values were determined by the Youden’s index (Youden’s index = sensitivity + specificity − 1). *p* ≤ 0.05 was considered to be statistically significant for all tests. The estimated values are presented with 95% confidence intervals (CIs). All of the analyses were performed using IBM SPSS Statistics 22.0 (IBM Corp., Armonk, NY, USA). 

## 4. Discussion

The main results of this study are twofold: first, in patients with severe influenza infection, and in those with bacterial co-infection, initial PCT and CRP levels on ICU-admission were not factors independently associated with the prognosis. Second, our findings revealed that, although respiratory co-infections due to gram positive bacteria were associated with higher levels of PCT compared with gram negative infections, none of the biomarkers were useful in predicting the bacterial etiology of infections.

The assessment of disease severity of patients with community acquired pneumonia (CAP) is important to optimize treatment and clinical management. Severity scores for CAP have limitations for clinical use, and they only have moderate sensitivity and specificity to identify mortality risk [21]. In this regard, measurements of serum biomarkers, such as PCT, CRP, interleukin-6, neopterin, and pro-adrenomedullin, are useful in the diagnosis and management of sepsis. In recent years, our knowledge has increased significantly, encompassing a broader approach that includes the application of genomics [22], proteomics, and metabolomics for a better understanding and management of infectious disease [23]. PCT and CRP are the most frequently used biomarkers in clinical practice, and that is why the present study focuses on both. Serum PCT increases with a greater severity of sepsis and organ dysfunction and the prognostic value of dynamic changes of PCT in patients with sepsis has been repeatedly demonstrated [24,25]. As opposed to these reports, the new international IDSA guidelines do not recommend the use of biomarkers for the initiation of antimicrobial treatment [26].

Our results concorded with most of the previous studies [27,28] showing that a single value of procalcitonin, at the time of ICU admission, cannot predict the mortality risk in critically ill septic patients. Two systematic reviews have been published, showing an association between procalcitonin on ICU admission and mortality, but the results must be interpreted very cautiously. Liu D et al. [29] analyzing 23 studies (*n* = 3994 patients), reported that elevated PCT concentrations were strongly associated with mortality in adult patients with sepsis, severe sepsis, and septic shock (RR, 2.60; 95%, CI 2.05–3.30). However, the diagnostic performance of a single initial PCT value was moderate for predicting sepsis mortality. More recently, the same author [30] presented a systematic review and meta-analysis evaluating the prognostic value of PCT in more than 6000 patients with pneumonia. The authors reported that high PCT levels were associated with an increased risk of mortality. Nevertheless, a few studies were conducted in critically ill patients, with a substantial heterogeneity.

Despite the fact that our results do not show a relationship between the initial value of PCT at admission and mortality in the ICU, PCT is a highly useful biomarker in the clinical management of septic patients, both its initial value and its trend during the following days. The PCT levels on admission are useful for the early diagnosis [6] and prompt treatment of community acquired pneumonia, and PCT trend is helpful during follow-up to monitor the evolution of the infection, the response to antibiotic treatment, and to predict outcome [24,25].

PCT and CRP have been proposed as able to identify different types of pathogens. Several studies have reported higher PCT concentrations in septic patients with proven gram negative bacteremia compared with patients with gram positive bacteremia or fungemia [11,12,31]. Nevertheless, most of the aforementioned studies were analyzing sepsis from different sources. Few studies have been conducted in a homogeneous population such as CAP. Tang JH et al. [32] in a recently published meta-analysis, reported that both PCT and CRP levels were higher in gram negative compared to gram positive and fungal infection. However, a selection bias, the limited number, the small sample size, and the high heterogeneity of published studies mean that the results should be interpreted with caution. Our results suggest a relationship between PCT levels and causative pathogens, but the discrimination power of this biomarker to identify the type of microorganism is low for gram positive and non-existent for gram negative isolations. The association of gram positive infection with high PCT concentrations is in agreement with another study that is based on non-bacteraemic respiratory infection [33]. It is also important to consider that systemic inflammation in sepsis is a complex process. Many studies [34] have reported different pathways of activation and the initiation of inflammatory cascades in regard to the etiology. The lower incidence of bacteremia in CAP and the high incidence in urinary tract infection probably contribute to the observed differences in PCT response seen with either gram positive or negative infections. 

To the best of our knowledge, our study is one of the largest published to evaluate the prognostic value and diagnostic accuracy of PCT in critically ill patients. The main strengths of this analysis are that it is conducted on a homogeneous population with a respiratory source of infection, a high number of critically ill patients from multiples sites, and excluding patients with high inflammatory status from a non-infectious origin, such as pancreatitis, trauma, or major surgery, which have been repeatedly reporting increased procalcitonin levels.

However, our study has some limitations. First, the blood samples were positive from only few patients, so it is difficult to compare results with most of the previous studies that are based on infections with positive blood cultures. Second, the study was conducted in critically ill patients with respiratory sepsis and septic shock; therefore, the results cannot be extrapolated to hospitalized patients with other sources of infection. Third, *Aspergillus* spp. isolation may correspond to colonization or infection, because a definitive diagnosis of invasive disease was not considered. However, the mortality rate in patients with *Aspergillus* spp. co-infection of 50%, similar to previously published results [35], suggests that it could be an infection rather than colonization.

In summary, a single initial serum measurement of the biomarkers (PCT and CRP) have a limited value to predict mortality in critically ill patients with influenza pneumonia. In this population, none of the biomarkers presented an adequate predictive value to determine the causative pathogen that is responsible for co-infection, so it should not be used to tailor empiric antimicrobial therapy.

## Figures and Tables

**Table 1:** Demographic and Clinical Characteristics of the patients enrolled in the study.

	Overall Population *n* = 1608	PVP *n* = 1186	BC *n* = 422	*p*-Value
Demographic Factors and Severity Scores				
Age (years)	56 (45–67)	55 (44–66)	60 (48–72)	0.001
Gender (male)	960 (59.7)	697 (58.8)	263 (62)	0.24
APACHE II score	17 (12–22)	16 (11–21)	19 (14–25)	0.001
SOFA score	6 (4–9)	6 (4–8)	7 (4–10)	0.001
Comorbidity				
COPD	328 (20.4)	77 (6.5)	31 (7.3)	0.45
Asthma	109 (6.8)	203 (17.1)	124 (29.4)	0.001
Chronic Heart Failure	210 (13.1)	149 (12.6)	61 (14.5)	0.34
Chronic Kidney Disease	146 (9.1)	109 (9.2)	37 (8.8)	0.77
Hematologic Disease	138 (8.6)	100 (8.4)	38 (9)	0.73
Pregnancy	240 (14.9)	168 (14.2)	72 (17.1)	0.16
Obesity ^a^	135 (8.4)	407 (34.3)	23 (5.5)	0.01
Laboratory Findings				
PCT (ng/mL)	0.81 (0.24–4.79)	0.6 (0.2–2.31)	4.35 (0.6–19.7)	0.001
CRP (mg/dL)	30.2 (14.8–113)	28.1 (13.3–109)	36.8 (20.4–118)	0.001
White Blood Cell Count (10^9^/L)	7.4 (3.8–12.4)	7.1 (4–11.6)	8.3 (2.9–15.2)	0.06
Complications				
Acute Kidney Failure	530 (32.9)	337 (28.4)	193 (45.7)	0.001
CRRT	215 (13.4)	140 (11.8)	75 (17.8)	0.002
Mechanical Ventilation	1318 (81.9)	974 (82.1)	342 (81)	0.65
Shock on Admission	920 (57.1)	634 (53.5)	284 (67.3)	0.001
MODS	1100 (68.4)	775 (65.3)	323 (76.5)	0.001
Clinical Outcomes				
MV (days)	10 (4–19)	10 (4–19)	10 (4–18)	0.85
ICU LOS (days)	10 (5–20)	10 (5–21)	10 (5–19)	0.73
ICU Mortality	382 (23.8)	264 (22.3)	117 (27.7)	0.02

Data are expressed as numbers (%) and medians (IQR). Abbreviations: PVP, primary viral pneumonia; BC, bacterial coinfection; IQR, interquartile range; APACHE, Acute Physiology and Chronic Health Evaluation; SOFA, sequential organ failure assessment; COPD, chronic obstructive pulmonary disease. PCT, procalcitonin; CRP, C-Reactive protein; CRRT, Continuous renal replacement therapy; MODS, Multiple organ dysfunction syndrome; MV, mechanical ventilation; LOS, length of stay; ICU, Intensive Care Unit. ^a^ Defined as body mass index >30 kg/m^2^.

## Data Availability

The anonymized database collected for the study by the SEMICYUC, and the data dictionary that defines each field in the set, will be made available to reviewers if they consider it necessary prior confidentiality agreement.

## Supplementary Materials

The following are available online at https://www.mdpi.com/article/10.3390/antibiotics10040350/s1, Supplemental Methods, Figure S1: Box plot of serum PCT and CRP concentration by the most frequent isolated microorganisms, Figure S2: Receiver operating characteristic curve for PCT to discriminate GP from GN pneumonia, Table S1: Cut-off levels for procalcitonin to predict GPC respiratory infection, Table S2: Characteristics of survivors and non-survivors in the study groups.
