# Supplementary material for: Prognostic Value of Procalcitonin and C-Reactive Protein in 1608 Critically Ill Patients with Severe Influenza Pneumonia
Source: Antibiotics (Basel). 2021 Mar 26;10(4):350. doi: 10.3390/antibiotics10040350 (PMC8066504; doi:10.3390/antibiotics10040350)
Supplement: Supplementary file 1 [file antibiotics-10-00350-s001.pdf]

antibiotics

MDPI

Article

Prognostic Value of Procalcitonin and C-Reactive Protein in 1608 Critically Ill Patients with Severe Influenza Pneumonia.

Raquel Carbonell [1,*], Gerard Moreno [1], Ignacio Martín-Loeches [2], Frederic Gomez-Bertomeu [3], Carolina Sarvisé [3], Josep Gómez [4], María Bodí [5], Emili Díaz [6], Elisabeth Papiol [7], Sandra Trefler [1], Mercedes Nieto [8], Angel Estella [9], María Jiménez Herrera [10], Pablo Vidal Cortés [11], Juan José Guardiola [12], Jordi Solé-Violán [13], and Alejandro Rodríguez [5].

1. Critical Care Department, Hospital Universitari Joan XXIII, Tarragona 43005, Spain; murenu77@hotmail.com (G.M.); sitrefler@yahoo.es (S.T.)
2. Department of Anaesthesia and Critical Care, St James´s University Hospital, Trinity Centre for Health Sciences, Multidisciplinary Intensive Care Research Organization (MICRO), Dublin 8, Ireland; drmartinloeches@gmail.com
3. Microbiology, Hospital Universitari Joan XXIII, Tarragona 43005, Spain; ffgomez.hj23.ics@gencat.cat (F.G.-B.); csarvise.tgn.ics@gencat.cat (C.S.)
4. Tarragona Health Data Research working group (THeDaR)- ICU Hospital Joan XXIII, Tarragona 43005, Spain; josep.goal@gmail.com
5. Critical Care Department URV/IISPV/CIBERES, Hospital Universitari Joan XXIII, Tarragona 43005, Spain; mbodi.hj23.ics@gencat.cat (M.B.); ahr1161@yahoo.es (A.R.)
6. Critical Care Department/CIBERES, Hospital Parc Taulí, Sabadell 08208, Spain; emilio.diaz.santos@gmail.com
7. Critical Care Department, Hospital Universitari Vall d'Hebron, Barcelona 08035, Spain; elisabeth_papiol@hotmail.com
8. Critical Care Department, Hospital Clínico San Carlos, Madrid 28040, Spain; mernietoc@gmail.com
9. Critical Care Department, Hospital de Jerez, Jerez de la Frontera 11407, Spain; litoestella@hotmail.com
10. Dean Nursing Faculty, Universitat Rovira i Virgili, Tarragona 43003, Spain; maria.jimenez@urv.cat
11. Critical Care Department, Complejo Hospitalario Universitario Ourense, Ourense 32005, Spain; pablomopvc@hotmail.com
12. Department of Pulmonary, Critical Care and Sleep Medicine, University of Louisville, Louisville 40202, USA; juan.guardiola@va.gov
13. Critical Care Department Hospital Universitario Dr. Negrín, Las Palmas de Gran Canaria 35010, Spain; jsolvio@gobiernodecanarias.org

* Correspondence: raquelviok7@gmail.com. Tel.: +34 687047670.

Citation: Carbonell, R.; Moreno, G.; Martín-Loeches, I.; Gomez-Bertomeu, F.; Sarvisé, C.; Gómez, J.; Bodí, M.; Díaz, E.; Papiol, E.; Trefler, S.; et al. Prognostic Value of Procalcitonin and C-Reactive Protein in 1608 Critically Ill Patients with Severe Influenza Pneumonia. *Antibiotics* **2021**, *10*, 350. https://doi.org/10.3390/antibiotics10040350

Academic Editor: Michele Bartoletti

Received: 22 February 2021
Accepted: 24 March 2021
Published: 26 March 2021

Publisher's Note: MDPI stays neutral with regard to jurisdictional claims in published maps and institutional affiliations.

Copyright: © 2021 by the authors. Submitted for possible open access publication under the terms and conditions of the Creative Commons Attribution (CC BY) license (http://creativecommons.org/licenses/by/4.0/).

1. Supplementary Online Content

1.1. Methods: Supplemental Methods

1.1.1. Data Collection and Validation

The data were collected using a paper CRF (case Report Form). CRF collect and record all protocol-required information, which is transcribed from patient source documents, such as hospital records and laboratory reports during the patient's participation in the study. Before being sent to the Study Coordinator (AR), these data were de-identified (not traceable to the patient) by removing the patient's name, medical record number, etc., and giving the patient a unique study number. We implemented a double data entry model for potential errors in real-time. Data were entered twice by two different Data Entry personnel based on the same set of data collected in the paper CRFs. All of the data were reviewed, and values that appeared incongruent or out of range were manually validated by confirming the accuracy of the data with the Study Coordinator (AR). The database was validated and cleaned before the statistical analysis and, finally, the study database was locked to prevent any further changes, and to ensure data consistency and integrity for the statistical reporting and analysis.

1.1.2. Study definitions

Shock was defined in accordance with the Surviving Sepsis Campaign guidelines (1); that is, patients in whom adequate fluid resuscitation and vasopressor therapy are unable to restore hemodynamic stability.

Acute Kidney injury (AKI) was defined according to Consensus Conference of the Acute Dialysis Quality Initiative (2).

References

1. Dellinger RP, Levy MM, Rhodes A, Annane D, Gerlach H, Opal SM et al. Surviving sepsis campaign: international guidelines for management of severe sepsis and septic shock: 2012. Crit Care Med 2013;41(2):580-637. https://www/doi/10.1097/CCM.0b013e31827e83af.
2. Bellomo R. Ronco C. Kellum J.A. et al. Acute renal failure—definition, outcome measures, animal models, fluid therapy and information technology needs: the Second International Consensus Conference of the Acute Dialysis Quality Initiative (ADQI) Group. Crit Care. 2004; 8: R204-R212

A. PCT (ng/ml)

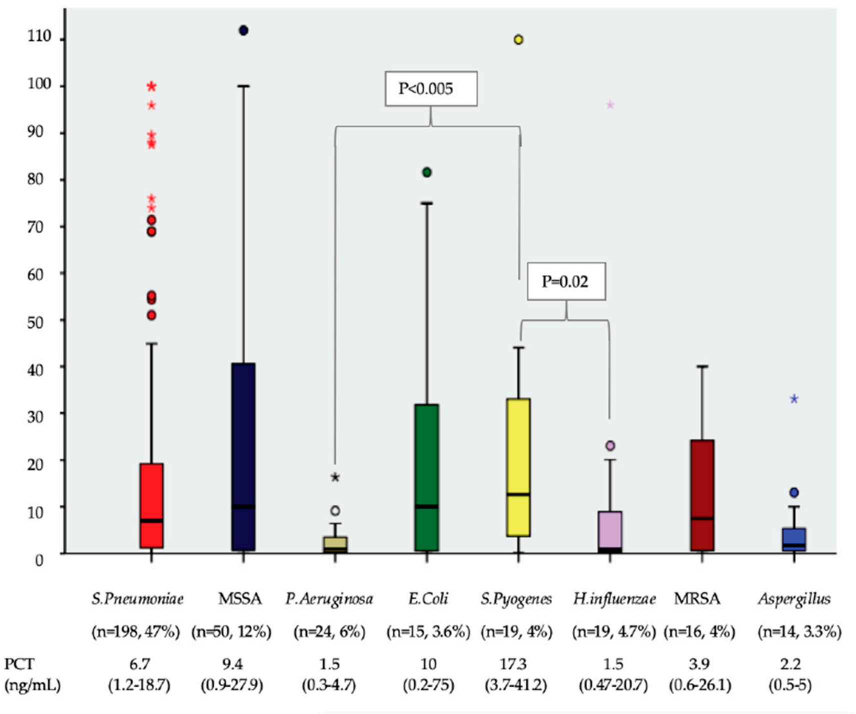

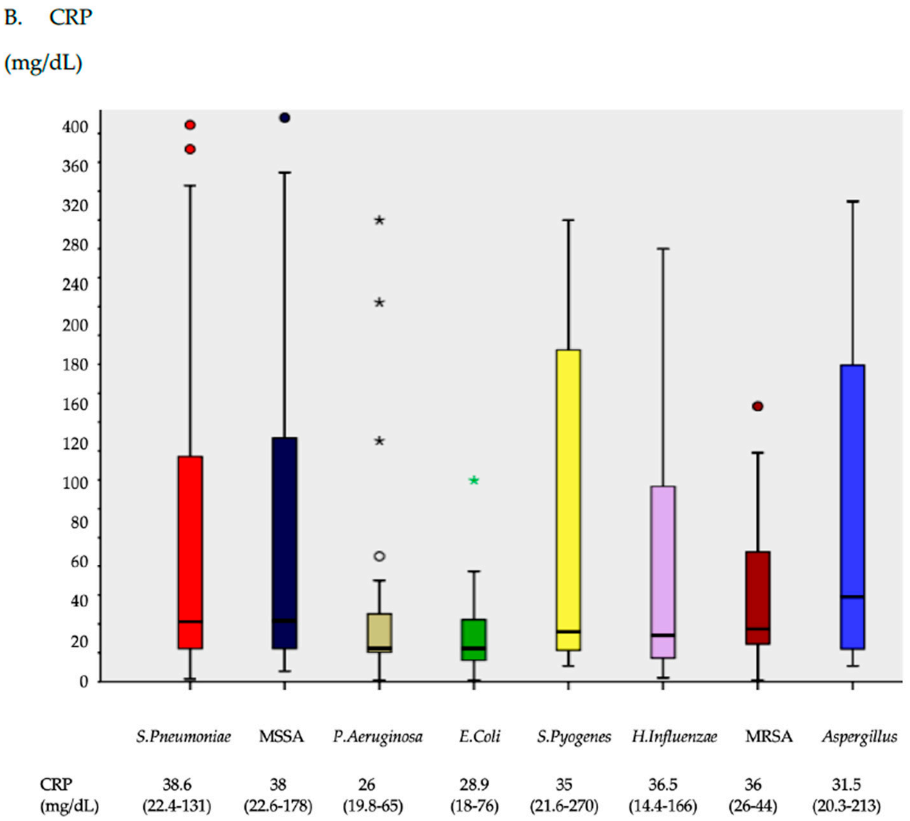

Figure S1. Box plot of serum PCT (**A**) and CRP (**B**) concentration by the most frequent isolated microorganisms.

The center of each box plot represents the median, with the box denoting the IQR, the whiskers representing 1.5 times the IQR, and dots showing outliers beyond the whiskers. Data are expressed as numbers (%) and medians (IQR). Abbreviations: PCT, procalcitonin; CRP, C-Reactive protein; MSSA, *Methicillin-sensitive Staphylococcus aureus*; MRSA, *Methicillin-resistant Staphylococcus aureus*; IQR (interquartile range).

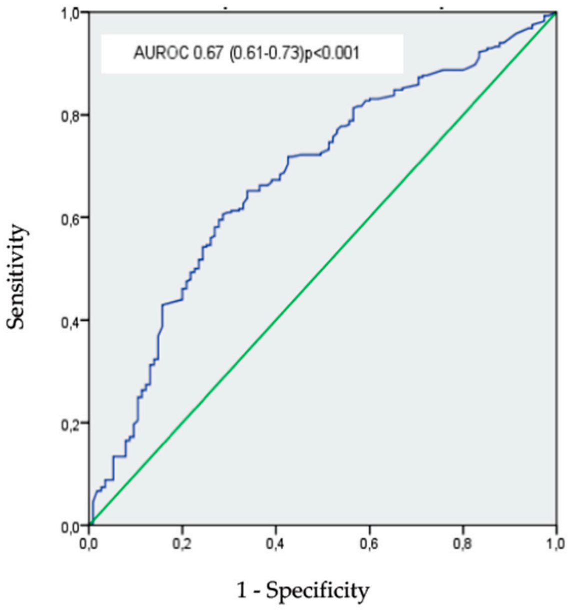

Abbreviations: PCT, procalcitonin; GPC, Gram Positive bacteria cocci; GNB, Gran Negative bacteria bacilli.

Figure S2. Receiver operating characteristic curve for PCT to discriminate GPC from GNB pneumonia.

Table S1. Cut-off levels for procalcitonin to predict GPC respiratory infection.

Cut-Off Levels for PCT (ng/mL)	Sensitivity (%)	Specificity (%)	PPV (%)	NPV (%)	LR+	LR-
>3.5	61.2	68.6	82.3	42.6	1.95	0.57
>5.5	54.4	73.7	83.2	40.5	2.07	0.62
>7.5	49.1	76.3	83.1	38.6	2.07	0.67
>10	42.7	83.1	85.7	37.8	2.53	0.69
>20	27	85.6	81.7	33.7	1.87	0.85
>30	17	84.7	72.3	31.2	1.14	0.95

Abbreviations: PCT, procalcitonin; PPV, Positive predictive value; NPV, Negative predictive value; LR+, positive likelihood ratio; LR-, negative likelihood ratio.

Table S2. Characteristics of survivors and non-survivors in the study groups.

	**PVP *n* = 1186**			**BC *n* = 422**		
	**Survivors (*n* = 922)**	**Non-survivors (*n* = 264)**	***P* value**	**Survivors (*n* = 305)**	**Non-survivors (*n* = 117)**	***P* value**
Age (years)	53 (43–64)	59 (50–68)	**0.001**	59 (49–71)	61 (48–74)	0.53
Gender (male)	534 (57.9)	163 (61.7)	0.26	183 (60)	80 (68.3)	0.13
APACHE II score	15 (10–20)	20 (15–26)	**0.001**	18 (13–24)	23 (18–28)	**0.001**
SOFA score	5 (3–8)	8 (5–11)	**0.001**	6 (4–9)	9 (6.2–12)	**0.001**
			**Comorbidity**			
COPD	157 (17)	146 (17.4)	0.88	88 (28.9)	37 (31.6)	0.59
Asthma	67 (7.3)	10 (3.8)	**0.043**	27 (8.9)	5 (4.3)	0.1
Chronic heart failure	109 (11.8)	40 (15.2)	0.15	34 (11.1)	27 (23.1)	**0.002**

Chronic Kidney Disease	73 (7.9)	36 (13.6)	**0.005**	24 (7.9)	13 (11.1)	0.29
Hematologic disease	54 (5.9)	46 (17.4)	**0.001**	21 (6.9)	17 (14.5)	**0.015**
Pregnancy	134 (14.5)	34 (12.9)	0.49	57 (18.7)	15 (12.8)	0.15
Obesity	87 (9.3)	26 (9.8)	0.79	16 (5.2)	7 (5.9)	0.77
			**Laboratory findings**			
PCT (ng/mL)	0.53 (0.19–2.1)	0.82 (0.3–2.8)	**0.001**	3.8 (0.5–17.37)	6.9 (0.93–28.4)	**0.039**
CRP (mg/dL)	18 (12.8–110)	28.6 (14.6–106)	0.48	37.4 (20.28–115)	33.2 (19–99.8)	0.23
White blood cell count (10^9/L)	7.1 (4.1–11.9)	7.2 (3.5–11.4)	0.65	8.8 (3.9–15.5)	6.8 (1.8–12.8)	**0.008**
			**Complications**			
Acute kidney failure	201 (21.8)	262 (99.2)	**0.001**	113 (37)	80 (68.4)	**0.001**
CRRT	54 (5.9)	86 (32.6)	**0.001**	32 (10.5)	343 (6.8)	**0.001**
Mechanical ventilation	722 (78.3)	252 (96.5)	**0.001**	229 (75.1)	115 (98.3)	**0.001**
Shock on admission	428 (46.4)	206 (78)	**0.001**	185 (60.7)	101 (86.3)	**0.001**
MODS	555 (60)	220 (83.3)	**0.001**	220 (72.1)	105 (89.7)	**0.001**
			**Clinical Outcomes**			
MV (days)	9 (4–18)	11 (5–20)	**0.03**	10 (5–19)	8 (3–17)	0.06
ICU LOS (days)	10 (5–20)	12 (5–21)	0.57	11 (5–20)	9 (3–17)	**0.019**

Data are expressed as numbers (%) and medians (IQR). Abbreviations: PVP, primary viral pneumonia; BC, bacterial coinfection; IQR, interquartile range; APACHE, Acute Physiology and Chronic Health Evaluation; SOFA, sequential organ failure assessment; COPD, chronic obstructive pulmonary disease. PCT, procalcitonin; CRP, C-Reactive protein; CRRT, Continuous renal replacement therapy; MODS, Multiple organ dysfunction syndrome; MV, mechanical ventilation; LOS, length of stay; ICU, Intensive Care Unit.

2. Complete list of GETGAG Investigators

2009–2018 H1N1 SEMICYUC Working Group investigators

Andalucía : Pedro Cobo (Hospital Punta de Europa, Algeciras); Javier Martins (Hospital Santa Ana Motril, Granada); Cecilia Carbayo (Hospital Torrecardenas, Almería);Emilio Robles-Musso, Antonio Cárdenas,Javier Fierro (Hospital del Poniente, Almería); Dolores Ocaña Fernández (Hospital Huercal – Overa, Almería); Rafael Sierra (Hospital Puerta del Mar, Cádiz); Mª Jesús Huertos, Mª Luz Carmona Pérez (Hospital Puerto Real, Cádiz); Juan Carlos Pozo Laderas , R. Guerrero, Juan Carlos Robles, Melissa Echevarría León,Alberto Bermejo Gómez (Hospital Reina Sofía , Córdoba); Enrique Márquez (Hospital Infanta Elena, Huelva); Manuel Rodríguez-Carvajal (Hospital Juan Ramón Jiménez, Huelva); Ángel Estella (Hospital del SAS de Jerez , Jerez de la Frontera); José Pomares, José Luis Ballesteros, Olga Moreno Romero (Hospital Universitario San Cecilio, Granada); Yolanda Fernández, Francisco Lobato, José F. Prieto, José Albofedo-Sánchez (Hospital Costa del Sol , Marbella); Pilar Martínez; María Victoria de la Torre; María Nieto, Estefanía Camara Sola (Hospital Vírgen de la Victoria , Málaga); Miguel Angel Díaz Castellanos, (Hospital Santa Ana de Motril, Granada); Guillermo Sevilla Soler, Carlos Ortiz Leyba (Clínica Sagrado Corazón, Sevilla); José Garnacho-Montero , Rafael Hinojosa, Esteban Fernández, (Hospital Virgen del Rocío , Sevilla); Ana Loza, Cristóbal León, Samuel González López (Hospital Universitario Nuestra Señora de Valme, Sevilla); Angel Arenzana,(Hospital Virgen de la Macarena, Sevilla), Dolores Ocaña (Hospital de la Inmaculada, Sevilla), Inés Navarrete (Hospital Virgen de las Nieves, Granada), Medhi Zaheri Beryanaki (Hospital de Antequera); Ignacio Sánchez, Manuel Pérez Alé (Hospital NISA Sevilla ALJARAFE, Sevilla); Ana Mª Poullet Brea (Hospital Quirón Málaga, Málaga); Juan Francisco Machado Casas (Complejo Hospitalario de Jaén, Jaén).

Aragón: Carlos Serón, Manuel Luis Avellanas, Arantxa Lander, S Garrido Ramírez de Arellano, MI Marquina Lacueva (**Hospital San Jorge, Huesca**); Pilar Luque; Elena Plumed

Serrano; Juan Francisco Martín Lázaro, Carlos Sánchez Polo, Isabel Gutiérrez Cia, Belén Jiménez Bartolomé, Carlos López Nuñez (**Hospital Lozano Blesa , Zaragoza**); Ignacio González, José Ignacio Tomás Marsilla, Clara Jaques Andrés, Pablo Gutiérrez Ibañes, Pilar Araujo Aguilar (**Hospital Miquel Servet, Zaragoza**); Jose Mª Montón (**Hospital Obispo Polanco, Teruel**); Paloma Dorado Regil **(Hospital Royo Villanova, Zaragoza)**

Asturias: Lisardo Iglesias, Carmen Pascual González, Brígida Quindós Fernández, Lorena Martín Iglesias, Lucía Viña Soria, Raquel Yano Escudero, Mª del Rosario Mtnez Revuelta (**Hospital Universitario Central de Asturias – HUCA-UCI 1, Oviedo**); Quiroga (**Hospital De Cabueñes, Gijón**); Águeda García-Rodríguez (**Hospital Valle del Nalón, Langreo**); Marta Martín Cuadrado, Ana Luz Balán Mariño **(Hospital San Agustín, Avilés);**

Baleares: Lorenzo Socias, Pedro Ibánez, Marcío Borges-Sa; A. Socias, Del Castillo A **(Hospital Son LLatzer, Palma de Mallorca**); Ricard Jordà Marcos, Cristina Muñoz (**Clínica Rotger , Palma de Mallorca)**; José M Bonell (USP**. Clínica Palmaplanas, Palma de Mallorca**); Ignacio Amestarán (**Hospital Son Dureta, Palma de Mallorca**); M.Angeles González López, Cecilia Vilanova Pàmies (**Hospital Mateu Orfila, Palma de Mallorca)**; José Ma. Bonell Goytisolo, José Antonio Morales Carbonero (**Hospital General de Muro, Palma de Mallorca)**; José Ma. Bonell Goytisolo, José Antonio Morales Carbonero (**Clínica Quirón Palmaplanas, Palma de Mallorca),** Rossana Pérez Senoff, Marta Generelo López de Medrano (**Hospital Comarcal de Inca, Palma de Mallorca).**

Canarias: Sergio Ruiz- Santana, Juan José Díaz, Catalina Sánchez Ramírez (**Hospital Dr Negrín ,Las Palmas de Gran Canaria**); Montse Sisón (**Hospital Doctor José Molina, Lanzarote**); David Hernández, Ana Trujillo, Luis Regalado, Sonia Rodríguez Fndez (**Hospital General la Palma, La Palma**); Leonardo Lorente, Judith Cabrera Rivero, Mª Luisa Mora Quintero (**Hospital Universitario de Canarias, Tenerife**); Mar Martín (**Hospital de la Candelaria, Tenerife**),Sergio Martínez, J.J. Cáceres, Manuel Sanchez Palacio, Marcos (**Hospital Insular de Gran Canaria**); D.García Rodríguez, María Ripoll Leria **(Hospital General de Fuerteventura, Fuerteventura)**;

Cantabria: Borja Suberviola, P. Ugarte (**Hospital Universitario Marqués de Valdecilla, Santander**);

Castilla La Mancha: Fernando García-López, Rafael Sánchez Iniesta (Hospital General, Albacete); Angel Álvaro Alonso, Antonio Padilla, Basi Martínez Palacios (Hospital General La Mancha Centro, Alcázar de San Juan); Mª Luisa Gómez Grande, Ma. Carmen Martín Rodríguez , Hasania Adbel-Hadi Álvarez, Alfonso Ambros Checa, Higinio Martín Hernández (Hospital General de Ciudad Real, Ciudad Real); Antonio Albaya, Alberto Silva Obregón, Carlos Marian Crespo, Carlos Armendariz Estrella, Carmen Benito Puncel, Eduardo Quirós Oyargue (Hospital Universitario de Guadalajara, Guadalajara); Alfonso Canabal , Luis Marina, Ismael López de Toro(Hospital Virgen de la Salud , Toledo); Almudena Simón (Hospital Nuestra Señora del Prado, Toledo); José María Añón (Hospital Virgen de la Luz, Cuenca)

Castilla y León: Juan B López Messa,(**Complejo Asistencial de Palencia, Palencia**), Mª Jesús López Pueyo , Ortíz María del valle, Sergio Ossa Echeverri (**Hospital General Yagüe, Burgos**); Zulema Ferreras, Juan C Ballesteros Herraez(**Hospital Universitario de Salamanca, Salamanca**); Santiago Macias, (**Hospital General de Segovia, Segovia**); José Ángel Berezo, Jesús Blanco Varela, Pablo Blanco Schweizer, Angela González Salamanca, Luis Tamayo Lomas (**Hospital Universitario Río Hortega , Valladolid**), Andaluz Ojeda Anzález, Ramón Cicuéndez Avila, Francisco Javier Pérez G (**Hospital Universitario, Valladolid);** Antonio Álvarez Terrero (**Hospital Virgen de la Concha, Zamora),** Fabiola Tena Ezpeleta, Christian Sala, Oliverio López **(Hospital Santa Bárbara , Soria);** Zulema Paez; Álvaro García **(Hospital Virgen Vega, Salamanca),** Demetrio Carriedo Ule, Miriam Riesco Crespo (**Complejo Asistencial Universitario de León-CAULE, León)**; Jesús Pino Rebolledo, Nicolás Hidalgo Andrés (**Hospital Campo Grande, Valladolid)**; Ana Carolina Caballero Zirena (**Complejo Asistencial de Zamora, Zamora);** Belén Román García, Juan

Bautista López Messa (**Hospital General Río Carrión, Palencia);** María del Valle Ortiz, Sergio Ossa Echeverri (**Hospital Universitario de Burgos-HUBU, Burgos)**

Cataluña: Rosa Mª Catalán (Hospital General de Vic, Vic); Miquel Ferrer, Antoni Torres, Catia Cilloniz (Hospital Clínic, Barcelona); Sandra Barbadillo Ansorregui(Hospital General de Catalunya – CAPIO, Barcelona); Lluís Cabré, Ignacio Baeza (Hospital de Barcelona, Barcelona); Assumpta Rovira (Hospital General de l'Hospitalet, L'Hospitalet);Francisco Álvarez-Lerma, Antonia Vázquez, Joan Nolla (Hospital Del Mar, Barcelona); Francisco Fernández, Joaquim Ramón Cervelló; Raquel Iglesia (Centro Médico Delfos, Barcelona); Rafael Mañéz , J. Ballús , Rosa Mª Granada(Hospital de Bellvitge, Barcelona); Jordi Vallés, Emili Díaz, Marta Ortíz , C. Guía, Ignacio Martín-Loeches (Hospital de Sabadell, Sabadell);Joaquim Páez (Hospital Dos De Mayo , Barcelona); Jordi Almirall, Xavier Balanzo, Estel Güell, Juan Carlos Yebenes (Hospital de Mataró , Mataró); Elena Arnau ,Marcos Pérez; César Laborda; Jesica Souto, Leonel Lagunes (Hospital Vall d'Hebron, Barcelona); Iñaki Catalán (Hospital Sant Joan de Déu , Manresa); Josep Mª Sirvent, Nerea López de Arbina, Anna Baró Serra, Adriana Sánchez, Silvia M. Cuenca (Hospital Josep Trueta , Girona); , Mariona Badía, Begonia Baseda-Garrido, Montserrat Valverdú- Vidal, Fernando Barcenilla, Mercedes Palomar, Xavier Nuvials (Hospital Arnau de Vilanova , Lleida); Pedro Garrido Benedicto (Hospital Sant Joan de Reus, Reus); Ferran Roche-Campo, MF Esteban, José Luna, Gaspar Masdeu Eixarch, Angels Pascual Diago (Hospital Verge de la Cinta, Tortosa); Juan Mª Nava , J González de Molina, Josep Trenado, Ricard Ferrer (Hospital Universitario Mutua de Terrassa, Terrassa); Zoran Josic, Montserrat Casanovas (Hospital de Igualada , Igualada); Francisco Gurri; Paula Rodríguez (Hospital Quirón, Barcelona) Alejandro Rodríguez, Laura Claverias, Sandra Trefler, María Bodí, Mónica Magret, Cristina Ferri (Hospital Universitario Joan XXIII, Tarragona); Rosa María Díaz (Hospital San Camil . Sant Pere de Ribes, Barcelona); Eduard Mesalles, Fernando Arméstar (Hospital Germans Trias i Pujol, Badalona); Diego de Mendoza, Carmen Lomas Fernández, José Julián Berrade (Hospital M. Broggi, Sant Joan Despí); Alfonso Bonet Saris, Marina Pechkova (Clínica Girona, Girona); Cristina Mora Jiménez (Hospital Sagrat Cor, Barcelona); Santiago Picos Gil (Clínica Diagonal)

Extremadura: Juliá-Narváez José, Manuel Robles Marcos, Vanessa Farje Mallqui, Mª. Angeles Santiago Triviño, Pablo Martínez García (**Hospital Infanta Cristina, Badajoz**), Alberto Fernández-Zapata, Teresa Recio, Abilio Arrascaeta , Mª José García-Ramos , Elena Gallego, Esther Saiz Rodrigo (**Hospital San Pedro de Alcántara, Cáceres**); Fernándo Bueno (**Hospital Virgen del Puerto, Plasencia)**; Mercedes Díaz, Noemí Gil Pérez, David López Hormigo **(Hospital de Mérida, Mérida);** Juan Diego Jiménez Delgado (**Hospital Don Benito, Villanueva de la Serena, Badajoz);** Pérez frutos , Rivera Pinna MJ **(Hospital Perpetuo Socorro)**

Galicia: Mª Lourdes Cordero, José A. Pastor, Luis Álvarez-Rocha, Alexandra Ceniceros Barros, Alejandra Virgós Pedreira (**CHUAC, A Coruña**); Dolores Vila, (**Hospital Do Meixoeiro , Vigo**); Carmen Fernández González (**C.H.U. de Ferrol, Ferrol)**; Javier Blanco Pérez, M Ortiz Piquer , (**Hospital Xeral - Calde, Lugo**); Eleuterio Merayo, Victor Jose López-Ciudad, Juan Cortes Cañones, Eva Vilaboy, José Villar Chao, Francisco Savira Cid López , Pablo Vidal Cortés, Marcos A. Pérez Veloso(**Complejo Hospitalario de Ourense , Ourense**); Eva Maria Saborido, Enrique Alemparte Pardavila, Ana Ortega Montes (**Hospital Montecelo, Pontevedra**); Raul José González, (**H. Miguel Domínguez, Pontevedra**); Santiago Freita , Enrique Alemparte; Ana Ortega (**Complejo Hospitalario de Pontevedra , Pontevedra**); Ana María López; Julio Canabal, Enrique Ferres **(Clinica Universitaria Santiago de Compostela, Santiago);** Javier Blanco Pérez, M Ortiz Piquer (**Hospital Lucus Augusti-HULA, Lugo**); Santiago Freitas Ramos, Lucas Lage Cendón, Vanesa Gómez Casal, Sabela Vara Adrio, Eva Menor Fernández, Susana González Prado (**H. Xeral-C.H.U. de Vigo, Vigo);** Antonio Varela Franco (**Hospital Vithas Nª Sª de Fátima, Vigo)**

La Rioja: José Luis Monzón, Félix Goñi (Hospital San Pedro, Logroño).

Madrid: Frutos Del Nogal Sáez, M Blasco Navalpotro, Ricardo Díaz Abad, José Luis Flordelis Lasierra (**Hospital Severo Ochoa, Madrid**); Mª Carmen García-Torrejón

(**Hospital Infanta Elena , Madrid**); César Pérez –Calvo, Diego López(**Fundación Jiménez Díaz, Madrid**); Luis Arnaiz, S. Sánchez- Alonso, Carlos Velayos, (**Hospital Fuenlabrada , Madrid**); Francisco del Río, Miguel Ángel González, Mercedes Nieto, Carmen Sánchez Cesteros **(Hospital Clínico San Carlos, Madrid**); María Cruz Martín , José Mª Molina (**Hospital Nuestra Señora de América, Madrid**); Juan Carlos Montejo, Mercedes Catalán (**Hospital Universitario 12 de Octubre, Madrid**); Patricia Albert, Ana de Pablo (**Hospital del Sureste, Arganda del rey**); José Eugenio Guerrero, María Zurita; Jaime Benitez Peyrat, Miriam Díaz Cámara (**Hospital Gregorio Marañón, Madrid**); Enrique Cerdá, Manuel Alvarez, Carlos Pey, Eva Manteiga Riestra, Concepción Martinez-Fidalgo (**Hospital Infanta Cristina, Parla**); Montse Rodríguez, Eduardo Palencia (**Hospital Infanta Leonor, Madrid**); Rafael Caballero, (**Hospital de San Rafael, Madrid**); Concepción Vaquero , Francisco Mariscal, S. García, Rico Cepeda (**Hospital Infanta Sofía, Madrid**); Nieves Carrasco, (**Hospital Universitario La Princesa, Madrid**); Isidro Prieto, A Liétor, R. Ramos, Rosario Cuadra Casas, Cruz Soriano Cuesta, Susana Sánchez Alonso (**Hospital Ramón y Cajal, Madrid**); Beatriz Galván, Juan C. Figueira, M. Cruz Soriano, Bélen Civantos Martín, Alejgandro Robles Caballero (**Hospital La Paz, Madrid**); P Galdós, Bárbara Balandin Moreno, Sara Alcántara Carmona (**Hospital Puerta de Hierro, Madrid**); Fernández del Cabo (**Hospital Monte Príncipe, Madrid**); Cecilia Hermosa, Federico Gordo (**Hospital de Henares, Madrid);** Alejandro Algora (**Hospital Universitario Fundación Alcorcón, Madrid**); Amparo Paredes, Teodoro Grau Carmona (**Hospital Sur de Alcorcón, Madrid**); JA Cambroner, Esther López Ramos, Yaiza Ortiz de Zárate (**Hospital Universitario Príncipe de Asturias, Madrid**); Sonia Gómez-Rosado, Margarita Mas Lodo, Nieves Franco Garrobo, Silvia Álvarez Hernández, Teresa Honrubia (**Hospital de Móstoles, Madrid**); Luis Miguel Prado López **(Hospital Sanitas La Zarzuela, Madrid)**; Esteban A, Lorente JA, Nin N, Carlos Jaramillo Sotomayor**(Hospital de Getafe, Madrid);** Luis Arnaiz **(Sanitas de Moraleja, Madrid)**; Esperanza Molero Silvero, Eduardo Morales Fdez. de la Reguera **(Hospital Central de la Defensa "Gómez Ulla", Madrid);** Rosa Mª de la Casa Monje, Fátima Martín Serrano **(Clínica Moncloa, Madrid);** Mª Victoria Trasmonte Martínez, M. Cruz Martín Delgado **(Hospital de Torrejón, Torrejón de Ardoz);**

Murcia: Sofía Martínez (**Hospital Santa María del Rosell, Murcia**); F. Felices Abad, Isabel Cremades Navalon, Martín Vigil Velis (**Hospital Universitario Reina Sofía, Murcia)**; Mariano Martínez, Domingo Martínez Baño, Enriqueta Andreu (**Hospital Universitario Virgen de la Arrixaca, Murcia**); Sergio Manuel Butí , Bernardo Gil Rueda, Francisco García (**Hospital Morales Messeguer, Murcia);** Noemí Llamas Fernández (**Hospital General Universitario Rafael Méndez, Lorca);** Luis Herrera Para, Alejandro Ortín Freire (**Hospital Universitario Santa Lucía, Cartagena);** Mª Rosa Nvarro Ruiz, C.R. Hernández Romero (**Hospital Los Arcos del Mar Menor, Murcia).**

Navarra: Enrique Maraví-Poma, I Jimenez Urra, Laura Macaya Redin, A Tellería (**Hospital Virgen del Camino, Pamplona**); Josu Insansti, (**Hospital de Navarra, Pamplona**). Noelia Artesero Garcia, Laura Macaya (Complejo Hospitalario Navarra-UCI A); Joaquín Lobo Palanco (Complejo Hospitalario Navarra UCI B)

País Vasco: Nagore González , Pilar Marco, Loreto Vidaur, Estibaliz Salas, Ruth Salaberría Udabe (**Hospital de Donostia, San Sebastián**); B. Santamaría, Tomás Rodríguez (**Hospital de Basurto, Bilbao**); Juan Carlos Vergara, Jose Ramon Iruretagoyena Amiano, Iratí Garrido Santos (**Hospital de Cruces, Bilbao**); Alberto Manzano, (**Hospital Santiago Apóstol , Vitoria**); Carlos Castillo Arenal (**Hospital Txagorritxu, Vitoria)**; Pedro María Olaechea, Higinio Martín Hernández **(Hospital Galdakao-Usansolo, Vizcaya);** Alejandro Martín López, Fernando Fonseca San Miguel (**Hospital Universitario de Alava-Santiago, Vitoria).**

Valencia: José Blanquer, Nieves Carbonell, José Ferreres Franco (**Hospital Clinic Universitari, Valencia**); Roberto Reig Valero , A. Belenger, Susana Altaba (**Hospital General de Castellón, Castellón**); Bernabé Álvarez –Sánchez, José Canovas Robles, Jaime Sánchez Francisco , Mar Ruiz Sánchez (**Hospital General de Alicante, Alicante**); Santiago Alberto Picos, Abilio Arrascaeta Llanes, Eugenio Herrero Gutiérrez, Alberto Fernández

Zapata (**Hospital Torrevieja Salud, Alicante**); Ángel Sánchez-Miralles, José Luis Antón Pascual (**Hospital San Juan, Alicante**); Juan Bonastre , M . Palamo, Javier Cebrian, José Cuñat, Mónica Gordón Sahuquillo (**Hospital La Fe , Valencia**); Belén Romero, Santiago Borrás Pallé, Javier de León Belmar (**Hospital de Manises, Valencia**); Rafael Zaragoza, Constantino Tormo, Susana Sancho Chinesta (**Hospital Dr Peset, Valencia**); Virgilio Paricio, (**Hospital de Requena , Valencia**); Asunción Marques, S. Sánchez-Morcillo, S. Tormo (**Hospital de la Ribera, Valencia**). J. Latour (**H.G Universitario de Elche, Valencia)**, M Ángel García, Manuel Palomo (**Hospital de Sagunto, Castellón)**, Francisco Tarín Royo, Pedro Manzano Hinojosa (**Hospital de Denia, Alicante)**; Mª Salomé Sánchez Pino (**Hospital Vega Baja, Alicante);** Concha Maragues Ribes, Rubén González Luis (**Hospital de la Plana, Castellón)**

Andorra: Antoli Ribas (Hospital Nuestra Señora de Meritxell, Andorra)
